# Predictors of loss to follow-up among children attending HIV clinic in a hospital in rural Kenya

**DOI:** 10.11604/pamj.2019.32.216.18310

**Published:** 2019-04-30

**Authors:** Winnie Mueni Saumu, Elizabeth Maleche-Obimbo, Grace Irimu, Rashmi Kumar, Christine Gichuhi, Bundi Karau

**Affiliations:** 1 Department of Paediatrics and Child Health, Chuka County Referral Hospital, Tharaka Nithi KE, Kenya; 2 Department of Paediatrics and Child Health, University of Nairobi, Nairobi, Kenya; 3 Department of Clinical Medicine and Therapeutics, University of Nairobi, Nairobi, Kenya; 4 Department of Internal Medicine, Kenya Methodist University, Nairobi, Kenya

**Keywords:** HIV infected, children, loss to follow up

## Abstract

**Introduction:**

African studies have reported high rates of loss to follow up (LTFU) among children in HIV care and treatment centres. Factors associated with LTFU may vary across populations. Few studies have been conducted among HIV infected children in care in rural areas of Kenya.

**Methods:**

this involved children aged less than 15 years on follow up at Kangundo Level 4 Hospital HIV clinic from January 2010 to December 2015. We obtained sociodemographic and clinical information from patient files and electronic databases. Univariate and multivariate regression analyses were conducted to identify factors predictive of LTFU.

**Results:**

a total of 261 HIV-infected children were followed up. The mean age was 10.0 years (IQR, 7-13) and median CD4 count of 582cells/ul (IQR 314-984). By December 2015, 171 children (65.5%) remained in active care, 32 (12.3%) transferred out, 13 (5%) died, while 45 (17.2%) were classified as LTFU. Out of the 45 children presumed as LTFU, we traced 44 out of the 45 children (98%) and found that their actual current status was as follows: 33 of the 44 children (75.0%) had dropped out of care (true LTFU). Factors strongly predictive of LTFU included low caregiver level of education (HR 2.3, 1.9-3.9, P = 0.001), WHO stage I and II at enrolment (HR 1.6, 1.4-2.1, P = 0.05).

**Conclusion:**

LTFU of HIV infected children was common with an incidence of 32.9 per 1000 child years and occurred early in treatment and risk factors included poverty, low caregiver education, male child and early HIV disease stage.

## Introduction

The prevalence of HIV in sub-Saharan Africa remains high, with an estimate of 34 million people infected. Of the 2.3 million HIV-infected children, about 90% are in sub-Saharan Africa making up 10% of People Living with HIV (PLHIV) in the region [1]. Although there has been an encouraging decline in the number of PLHIV in Kenya, about 1.6 million people are still infected, with children below 15 years making up 12% [2]. Anti Retroviral Therapy (ART) coverage in Kenya has significantly expanded over the past decade with 78.4% of those requiring ART accessing ART but coverage among children remains low with only 32% of those needing ART receiving it [2]. The long-term success of ART in children depends on adherence to treatment and follow-up. This is critical in children, as they will require life-long therapy [3] without which HIV disease progresses more rapidly than in adults. As ART coverage expands, a rise in loss to follow-up (LTFU) has been observed in many ART programmes in Africa and is notably worse among children [4]. LTFU is, therefore, a major impediment to successful implementation of HIV care and treatment programmes in sub-Saharan Africa, with an estimated 20-40% of patients being lost to follow up [4]. LTFU is associated with increased risk of ART failure, morbidity, mortality and hospitalizations [5]. LTFU among children in Kenya has been largely evaluated in research settings in tertiary referral hospitals and their catchment areas. In western Kenya, 50% of children were found to have ever missed an appointment [6]. According to Kenya National Aids and STI Control Programme [7], 18% of children aged below 10 years and 32% of adolescents are LTFU. About 18% of children enrolled in ART programmes in western Kenya will be LTFU [8]. Several sociodemographic and clinico-immunological factors have been reported to contribute to LTFU among children in ART programmes. Factors associated with LTFU may differ from population to population. A better understanding of these factors will help to develop targeted interventions to improve retention and predict the risk of LTFU among newly enrolled children so as to institute proper measures on initial contact. The aim of the present study was to determine the factors predictive of LTFU among children enrolled in an HIV care and treatment programme in a rural hospital in eastern Kenya.

## Methods

**Study design:** a cross-sectional was carried out at Kangundo Level 4 Hospital, which runs an HIV clinic with a total enrolment of 3500 patients with 300 being children below 15 years of age. LTFU was defined as patients who did not return for care or treatment for a period of 3 months or more since their most recent documented appointment date despite repeated phone contact.

**Data collection:** the study was approved by Kenyatta National Hospital/University of Nairobi Ethics and Review Committee. Physical files of all children under care in the HIV clinic between January 2010 and December 2015 were retrieved from the records office by the records officer assisted by the peer counsellors. Case record forms were used to capture sociodemographic characteristics such as age, gender, caregiver of the child, caregiver education level, HIV status of caregiver, whether biological parents were alive and economic status of parents or caregivers. Clinical characteristics captured included baseline CD4 count, WHO stage at enrolment, ART regimen initiated and history of opportunistic infections. Children were categorized as active in care, lost to follow-up, dead or transferred out. Those lost to follow up were traced physically to determine their true status of follow up as either self-transferred out, dead or lost to follow up.

**Data analysis:** data from the questionnaires was entered into SPSS Version 23.0, IBM. Descriptive statistics of patients and caregiver characteristics were determined. Period prevalence of LTFU and incidence rate were calculated. Univariate predictors of LTFU versus retention in care were then determined using Chi-square, Fisher's exact test and reported as hazard ratios with significance set at 5% (P<0.05). A multivariate logistical regression model was constructed.

## Results

Between January 2010 and December 2015, a total of 261 children were found to be on follow-up at the HIV clinic at Kangundo Level 4 Hospital. The mean age of the children was 9.98 yrs, (median 10 years (Interquartile range 7-13) with 57.1% (149) and 42.9% (112) males and females respectively. Table 1 summarizes the baseline sociodemographic characteristics of the children. The median CD4 count among the entire cohort of enrolled children was 582 cells/ul. Majority (84.7%) were on ART. The baseline clinical characteristics of the children are summarized in Table 2.

**Table 1:** baseline socio-demographic characteristics of children

Characteristic	Frequency	%
**Age Group**		
<2 yrs	5	1.9
2-5 years	37	14.1
6-10	73	28
11-14	146	56
Median age	10	IQR(7-13)
**Gender**		
Male	149	57.1
Female	112	42.9
**Child in school**		
Yes	239	91.6
No	20	7.7
Missing data	2	0.8
**Biological mother alive**		
Yes	131	50.2
No	127	48.7
Missing information	3	1.1

**Table 2:** baseline clinical characteristics of the entire cohort of enrolled children

Clinical characteristic	All children enrolled in the clinic	
	N	%
**WHO stage at enrolment**		
Stage I	70	26.8
Stage II	115	44.1
Stage III	69	26.4
Stage IV	2	0.8
**Baseline CD4 count**	**Median**	**IQR**
	636.5	314-984
**Weight: WAZ**		
0 to -1	107	39.9
<-1 to -2	126	47.0
<-2 to -3	33	12.3
<-3	2	0.8
**Whether on ART**		
Yes	221	84.7
No	33	12.6
**First line ART regimen**		
Zidovudine-based	88	23.6
Abacavir-Based	122	46.7
Stavudine-Based	6	2.3
Tenofovir-Based	7	2.7

**Status of follow-up:** status of follow-up was categorized as; those in active care, dead, transferred out, or loss to follow-up. Over the study period, 65.5% (171) of the children continued under active care, while 17.2% (45) satisfied the criteria of loss to follow-up. Table 3 below summarizes the status of follow-up.

**Table 3:** child retention status at time of study (per medical record abstraction)

Characteristic	Frequency or Median	(%) IQR
**Retention Status**		
Active	171	(65.5)
Transferred out	32	(12.3)
Dead	13	(5)
LTFU	45	(12.3)
**Duration in care in months**		
Active	48	24-60
Transferred out	29	18-36
Dead	30	27-34
LTFU	8	4-34
Among LTFU-Time to LTFU	N= 45	
<6 months	16	(35.5)
6 months-1 year	9	(20)
1-3 years	12	(26.7)
>3 yrs	8	(17.8)
Incidence of Loss to Follow up	44.5 per 1000 child years	

**Predictors of loss to follow-up:** after univariate analysis, male children (p = 0.025), children whose caregivers had low level of education (p = 0.001), and child not on ART (p = 0.0001) were among the factors found to be significantly correlated with LTFU. Below is a tabular demonstration of the factors associated with loss to follow up (Table 4). After construction of a multivariate logistical regression model we found that low caregiver level of education was most predictive of LTFU (p = 0.001). Table 5 illustrates the logistical regression model for the selected independent factors associated with LTFU.

**Table 4:** univariate analysis of the factors associated with Loss to follow up

Characteristic	Lost to Follow-up Freq (%)		Not Lost to Follow-up Freq (% )		Unadjusted OR (95% CI) and P-value
**Gender**					
Male	26	(17.5)	123	(82.5)	
Female	19	(16.8)	93	83.2	
**Caregiver level of education**					2.50 (1.05-4.55), 0.01
None &Primary	34	(23.4)	111	(76.6)	
Secondary & above	11	(13.3)	72	(86.7)	
**WHO stage at enrolment**					1.88 (0.71-3.22), 0.03
Stage I and II	36	(18.8)	156	(81.2)	
Stage III and IV	7	(9.7)	65	(90.3)	
**Primary caregiver**					1.12 (1.08-3.33), 0.05
Parents	28	(17.9)	128	(82.1)	
Relatives	17	(16.1)	88	(83.9)	
**Caregiver source of income**					1.66 (1.182-1.912), 0.039
Informal employment/farmer	42	(19.5)	183	(80.5)	
Formal employment	1	(12.5)	7	(42.5)	
**Child on ART** Yes		(10.4)	198	(89.6)	5.83. (4.23-9.76), 0.01
No		(57.5)	14	(42.5)	
**History of regimen switch**					1.4 (1.0-1.6), 0.05
Yes	3	(6.5)	43	(93.5)	
No	38	(18.4)	168	(81.6)	

**Table 5:** multivariate model of predictors of loss to follow up

Independent variable	Adjusted OR (95% CI)	P-Value
Male gender	1.22 (1.08-2.63)	0.02
Low caregiver education level	2.30 (1.87-3.94)	0.001
WHO Stage I & II	1.62 (1.44-2.09)	0.05
Child not on ART	4.70 (4.43-5.97)	0.03

**Status of follow up after tracing:** out of the 45 children who were lost to follow up, we were able to trace and know the status of 44 of them. 33 (75.0%) of the 44 had dropped out of care (True loss to follow up), while 6 (13.6%) were dead and 5 (11.4%) had transferred to other facilities without referral. The true period prevalence of LTFU over the study period was found to be 12.6% (n = 261). The true Incidence of Loss to Follow- up was 32.9 per 1000 child years.

## Discussion

A total of 261 children were followed up during the study period with a median age of 10 years, with 57.1% and 42.9% being male and female children respectively. The child's primary caregiver was the mother in 51.3% of the cases, followed by grandmother in 19.5% of cases. In 41.8% of the cases studied, the biological mother was deceased, while the father was deceased in 48.7% of the cases. This means that a substantial number of the children are under the care of grandmothers and relatives, who often have poor socioeconomic and education status, fuelling loss to follow-up. This has been identified as one of the challenges in the provision of HIV care in resource-limited setting [9].

**Magnitude of loss to follow up:** the true cumulative incidence was 32.9 per 1000 child years of LTFU over the study period and the periodic prevalence was 12.6%. This magnitude of LTFU of 12.6% is in agreement with findings from previous studies conducted in sub-Saharan Africa. About 21% of patients in HIV programmes are LTFU in Africa [10]. LTFU differs radically across populations because authors use different definitions. For instance, a Ugandan study reported 50.1% of children were LTFU [11], while a South African study reported a LTFU rate of 50.2% [12]. Different definitions of LTFU carry the inherent risk of misclassification of active patients as LTFU. Some studies cite absence of 1 week from a scheduled appointment [13], absence of 2 weeks after an appointment date [14], while other use 6 weeks of missed appointment [15]. We employed the WHO definition of LTFU as more than 90 days absence from the missed clinical or drug pick-up appointment without any follow up visit [16], a definition also used by NASCOP, Kenya. In Kenya, previous studies have focused on HIV programmes in referral hospitals or urban centres. Our study is among the very few looking at LTFU in rural settings. In a study conducted in Western Kenya, 14.2% of children were LTFU. For children already initiated on ART, 14.1% dropped out of care [8]. This shows that the magnitude of LTFU in this referral setting does not significantly differ from our study in a rural setting. It has been reported that 10-14% of children on ART get lost to follow up in HIV care programmes in Africa [8].

**Factors predictive of loss to follow up:** male children were more likely to be lost to follow up compared to female children. Our finding is similar to what has been reported by researchers in South Africa [17], Uganda [18] and the KIDS-ART-LINC collaboration study [19]. Another study done in Uganda reported a higher incidence of LTFU among female children compared to male, although the difference was not statistically significant [20]. The reasons for the gender difference in LTFU have not been elucidated in the literature and may vary across ethnicities. There is need to identify issues facing each of the genders and address them specifically. Low caregiver level of education was associated with increased LTFU. A similar trend has been reported in a Brazilian study conducted in Pernambuco [9]. In the current study setting, most of the caregivers were grandmothers, as the children were orphaned. These caregivers had little or no formal education and are unlikely to understand the importance of adherence to clinic appointments. Further, caregivers without formal education are more likely to believe in faith healing and use of traditional medicine, increasing the likelihood of LTFU [21].

Our study found that 67.4% of the children LTFU were in WHO stages I and II. Further, WHO stage I and II was positively correlated with LTFU, with children in this stage 1.88 times more likely to be LTFU compared with those in stage III and IV. These findings are supported by a study conducted in Mozambique [22]. This may be explained by the fact that caregivers have the notion that children with early HIV disease are not sick, and therefore don't adhere to clinic appointments. However, several studies have attributed LTFU to severe immunosuppression. In the KIDS-ART-LINC study [19], there was a positive correlation between LTFU and severe clinical status defined as WHO stages III and IV. Massavon *et al.* [23] found that children in stage III and IV were more likely to be LTFU. Surprisingly, children who had biological parents as caregivers were found to be more likely to be LTFU. A similar trend was reported in the study, wamepotea in western Kenya, where orphans were less likely to be LTFU [8]. Many studies have also reported that in cases where the biological mother is the primary caregiver, there is a high likelihood of LTFU [17, 18]. A possible explanation is that in most cases, the mother is HIV positive and may be in denial, too ill to honour clinic appointments, or suffering from guilt. Children whose caregivers were in informal employment were found be more likely to be LTFU in the current study. This may be related to the financial constraints of paying for transport from home, as has been found in previous studies [3]. A study done in western Kenya found that provision of food to child in the HIV clinic appeared to reduce the incidence of LTFU [24]. This is because most of the caregivers of these children have difficulties providing food and basic amenities to these children, partly because of the cost of other opportunistic infections the child may have.

We found that children not on ART were more likely to be LTFU. This is in agreement with the findings from other studies that have shown that LTFU in the pre-ART group is significantly higher than post-ART initiation group [25]. In our cohort, majority of children were LTFU in the immediate period before 6 months after enrolment. It is possible that before initiation of ART, children and caregivers are not counselled properly, hence are more likely to be LTFU. Those children being started on ART are counselled thoroughly hence are more likely to adhere to clinic appointments. For the children LTFU, 44 caregivers were successfully traced. Out of the 44 children traced, 33 (71.1%) were reclassified as true LTFU, 13.3% were deceased, while 13.3% had transferred to other facilities. This mirrors the findings of the wamepotea study in western Kenya, where 16% of the traced children were found to be deceased [23]. In this study, 11% were found to have transferred to other clinics. The finding that some children initially classified as LTFU may be deceased means that the mortality rate due to HIV may be higher than thought. A study in Malawi found that HIV patients LTFU had generally higher mortality rate than the general population [26]. This underscores the need for active tracing of these children.

## Conclusion

Children from lower socio-economic status families whose caregivers are of low education and who have early HIV disease at entry of care and not on ART are more likely to drop out of care. The caregivers with low education level should be given more counselling to ensure their understanding of the importance of adhering to clinic appointments.

### What is known about this topic

- It is already known that there is high prevalence of loss to follow-up among children enrolled in HIV care and treatment programmes.
- Factors associated with loss to follow-up have been described in many studies and have been noted to vary across populations and countries.

### What this study adds

- This study elucidates factors associated with loss to follow-up in a predominantly rural population;
- It reveals that true loss to follow-up should be declared after active tracing of children who drop out of care.
